# International survey on immunological diagnostics in children with sepsis

**DOI:** 10.1007/s44253-026-00120-w

**Published:** 2026-05-29

**Authors:** Nina Schöbi, Philipp K. A. Agyeman, Vanessa Sancho-Shimizu, Johannes Trück, Julie C. Fitzgerald, Luregn J. Schlapbach

**Affiliations:** 1 Division of Pediatric Infectious Disease, Department of Pediatrics, Bern University Hospital, Inselspital, University of Bern, Bern, CH-3010 Switzerland; 2 Department of Infectious Disease, Imperial College London, London, UK; 3 Centre for Paediatrics and Child Health, Imperial College London, London, UK; 4 Divisions of Allergy and Immunology and the Children’s Research Center, University Children’s Hospital Zurich, University of Zurich, Zurich, Switzerland; 5 Division of Pediatric Critical Care Medicine, Department of Anesthesiology and Critical Care, The University of Pennsylvania Perelman School of Medicine, Philadelphia, PA USA; 6 Department of Intensive Care and Neonatology, and Children’s Research Center, University Children’s Hospital Zurich, University of Zurich, Zurich, Switzerland; 7 Child Health Research Centre, The University of Queensland, Brisbane, QLD Australia

**Keywords:** Child, Infection, Primary immunodeficiency, Rare disease, Invasive infection, Sepsis

## Abstract

**Background:**

Sepsis is a leading cause of death and disability in children and it affects in up to 50% previously healthy children who have access to health care. Undiagnosed inborn error of immunity, considered to be rare at population level, may underlie host immune dysregulation and result in exceptionally high risk for life-threatening infections.

**Methods:**

From May 2023 to March 2024, physicians involved in the care of children with sepsis were questioned regarding their follow-up investigation practice in paediatric patients after community-acquired sepsis. This survey was distributed through three international societies.

**Results:**

A total of 168 responses from 50 countries were analysed. The majority (82%), reported to have more than 10 years of experience. More than half (53%) stated to work in Paediatric Infectious Diseases. Major variability across respondents was observed in the approach to different scenarios of patients with sepsis. Severity of presentation, and unusual presentations increased the likelihood of ordering immunological investigations. Only 7% reported to have access to a standardised sepsis specific guideline to immunologically investigate paediatric patients after sepsis.

**Conclusion:**

The results of this survey highlight a broad range of practices surrounding immunological investigations of children with sepsis. A consensus-based algorithm for standardised immunological and genetic testing in paediatric patients after sepsis could guide practice and inform future studies in the field.

**Supplementary Information:**

The online version contains supplementary material available at 10.1007/s44253-026-00120-w.

## Introduction

Sepsis, a syndrome with life-threatening organ dysfunction associated with infection [1], is a leading cause of death and disability in children [2]. Globally, sepsis accounts for approximately three million childhood deaths per year [3]. In high-income countries with high immunisation coverage, almost half of paediatric sepsis deaths occur in previously healthy infants with appropriate access to health care [4–7]. Over the past decade, it has been increasingly recognised that immune dysregulation in patients with sepsis can contribute to uncontrolled or ineffectively controlled infection but also result in an overactive inflammatory response, both significantly impacting patient outcomes [8, 9]. However, these mechanisms remain poorly understood in children [10]. Notable to paediatric age groups, undiagnosed inborn errors of immunity (IEI) may underlie immune dysregulation and result in an exceptional vulnerability to life-threatening infections in apparently healthy hosts [11–15]. Undiagnosed underlying immunodeficiency may expose survivors to risks of reoccurrence, or may lead to missed opportunities of diagnosing cases in family members and reduce morbidity and mortality by counselling and implementing secondary prevention [12]. Moundir et al. reported 13% of potentially causal genetic variants in previously healthy children with sepsis, whereas Kernan et al. reported up to 44.5% of IEI-linked variants in paediatric patients with sepsis admitted to intensive care [14, 15]. In a national population-based study, Borghesi et al. found 2% of exact variants for IEI associated with an increased risk for bacterial infection in children with community-acquired sepsis, which is higher than the estimated prevalence at population level [13, 16]. Currently, however, there are no guidelines to advise on immunological or genetic investigations in children after a sepsis episode.

With the present survey, we sought to understand the current practice of physicians involved in the care of children for investigating children after sepsis in relation to their immune competence on which topic no prior international survey exists.

## Methods

A group of national experts in sepsis and healthcare professionals involved in the Swiss Pediatric Sepsis Study together with an international researcher in this field (VSS) developed the survey. The survey was first trialed by all authors and was sent thereafter for external review by a second international expert of sepsis (JCF). The survey was endorsed by the European Society of Paediatric Infectious Diseases (ESPID), the Pediatric Infectious Diseases Society (PIDS), and the Australian and New Zealand Intensive Care Society Paediatric Study Group (ANZICS-PSG). From May 2023 to March 2024, an invitation to answer the online survey, hosted on the Research Electronic Data Capture (REDCap©) platform, was distributed including reminders through these three societies which were selected to ensure international and interprofessional coverage, including representatives of the important stakeholders involved not only to guide acute phase management of sepsis but also determine follow-up assessments based on identifying patients with typical versus atypical courses. No incentive was offered to respondents to complete the survey. Sepsis was defined as children with infection and associated organ dysfunction (this included children with septic shock and children with other infection-associated organ dysfunctions). This definition was part of the introductory part of the survey.

### Data set

The survey instrument is provided in the online supplement. The survey consisted of multiple choice, 5-point Likert scale questions, and clinical vignettes asking whether and how children after an episode of sepsis were investigated. Respondents were eligible if they were involved in paediatric care, i.e., treating children as a medical doctor or contributing to patient care decisions through multidisciplinary team meetings. The first survey part covered aspects of the respondents’ professional attributes, e.g., size of the hospital, and role at the hospital. Next, general questions regarding important risk factors for the presence of an IEI but also accessibility to immunological investigations were examined. The survey then provided six case vignettes describing different clinical scenarios, spanning from uncomplicated bacteraemic pneumonia, Gram negative septic shock on PICU, meningitis with neurocognitive sequelae, repeated invasive bacterial infection to sepsis with fatal outcome. We asked respondents to state whether, and, if yes, which, investigations they would routinely perform in such a case.

### Data analysis

Data was extracted from REDCap© into Microsoft Excel^®^. Data were expressed as numbers and/or percentages for categorical variables. We excluded responses that contained no completed questions beyond respondents’ background information—or even fewer entries—resulting in no available data on immunological investigation practices. Because mailing lists may contain duplicate email addresses, inactive members, or members without registered email contacts societies were unable to provide reliable figures for calculating reliable response rates. We calculated proportions and reported results based on the number of respondents who answered a particular question. Data was analysed using R software package, version 4.0.3 (Vienna, Austria; https://www.R-project.org/).

### Ethical considerations

According to the Swiss National Research Ethics Committee, ethical approval was not required for a survey involving healthcare physicians. Participation in the survey was voluntary. No identifying personal information was collected. Participants provided consent by responding to the survey.

## Results

### Characteristics

**Fig. 1:**
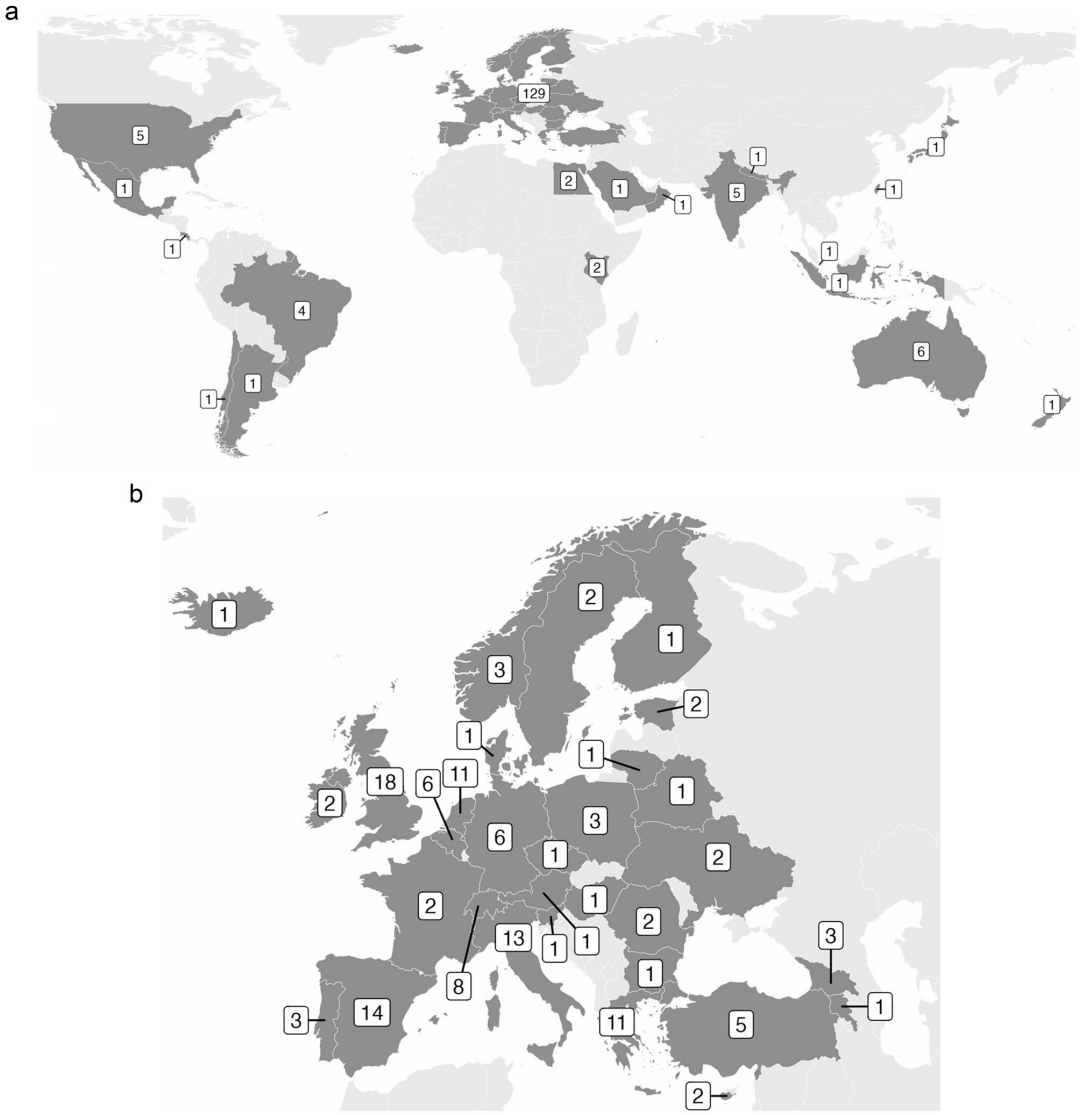
The map shows the number of respondents practicing in each country

A total of 223 healthcare professionals started the survey, 55 had to be excluded because of incomplete responses. Of those, 29 did not answer a single question, 23 answered the questions regarding their personal characteristics but did not answer any questions on immunological follow-up practice in patients after community-acquired sepsis, and 3 reported not to be treating children as a medical doctor or contributing to patient care decisions. A total of 168 healthcare professionals from 50 countries completed the survey, 129/168 (77%), were respondents from Europe (Fig. 1a/b). The majority (138/168, 82%) of the respondents had 10 or more years of experience, and 127/168 (76%), classified themselves as consultants. More than half reported to work in Paediatric Infectious Diseases, 89/168 (53%), followed by General Paediatricians, 31/168 (18%), Paediatric Immunologists, 19/168 (11%), and Paediatric Intensivists, 9/168 (5%). Most of the respondents, 135/168 (80%), stated to work at least partially in a tertiary centre, and 100/168 (60%) to have access to a dedicated immunological service on-site. Table S1.

Eleven of 168 (7%) respondents reported using a standardised, sepsis-specific guideline to immunologically investigate paediatric patients after sepsis, 43/168 (26%) reported using an institutional guideline, not sepsis-specific, and 114/168 (68%) reported not having any guideline at their centre. Almost all, 140/146 (96%) stated that an algorithm for a standardised immunological work-up for paediatric patients after sepsis would be helpful 5/146 (3%) did not know, and 1/146 (0.7%) disagreed.

### Immunological investigation practices

Decision on immunological testing was reported to be taken by Immunologists, Infectious Diseases Specialists, and other physicians in 47%, 24%, and 27%, respectively and the majority (43%) reported to order immunological testing 11 to 50 times per year in children after community-acquired sepsis, see Table S1.

**Fig. 2:**
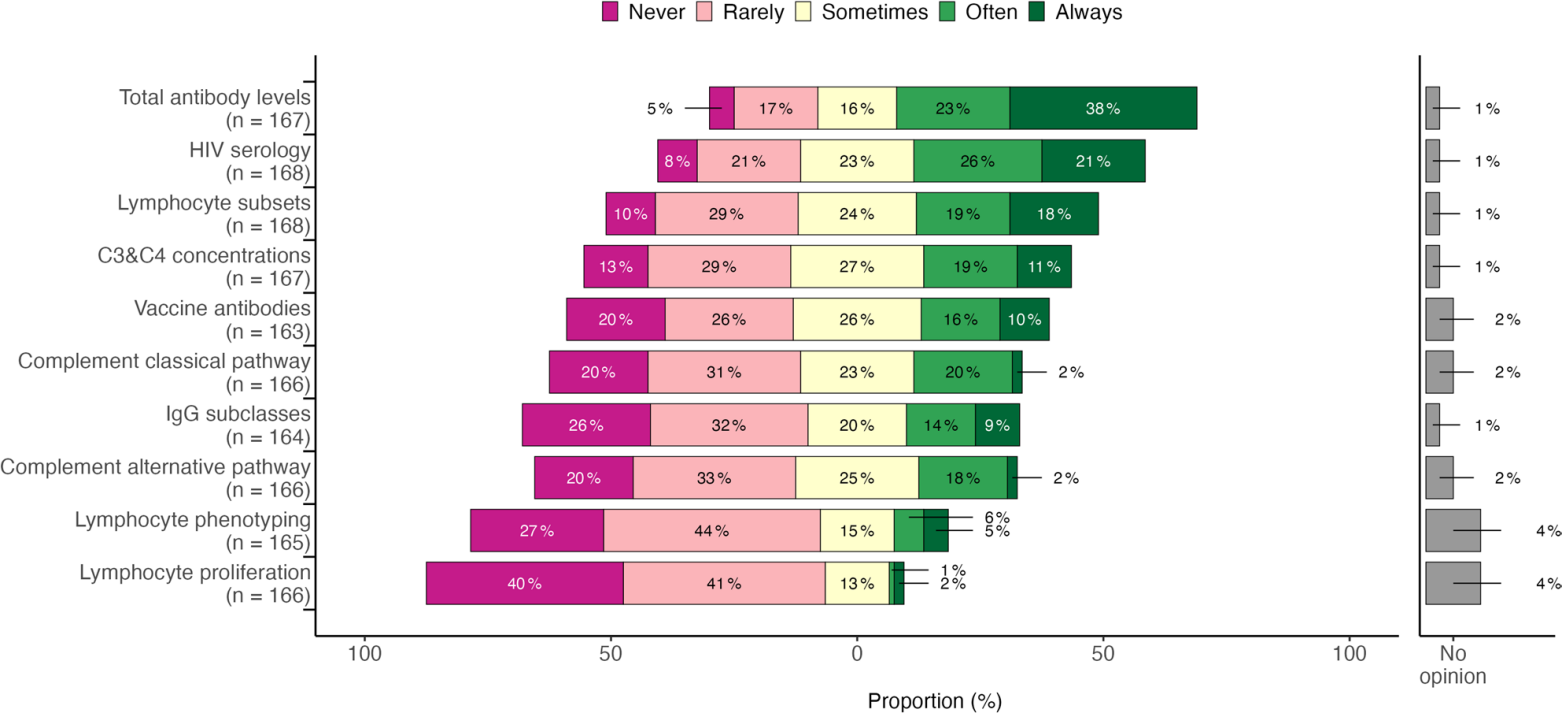
The graphs show, for each test, the proportion of respondents who reported almost always, often, sometimes, rarely, or never performing the test in children (> 1 month to 18 years of age) presenting with community-acquired sepsis in the absence of known major comorbidities (e.g. iatrogenic immunosuppression, central venous access, chromosomal syndromes, major chronic handicap)

More than half of the respondents (61%) reported to measure almost always or often total antibody levels and almost half (47%) to order HIV serology in paediatric patients after sepsis, as compared to 37% almost always or often measuring lymphocyte subsets (Fig. 2). Additional testing was reported to be ordered more rarely.

**Fig. 3:**
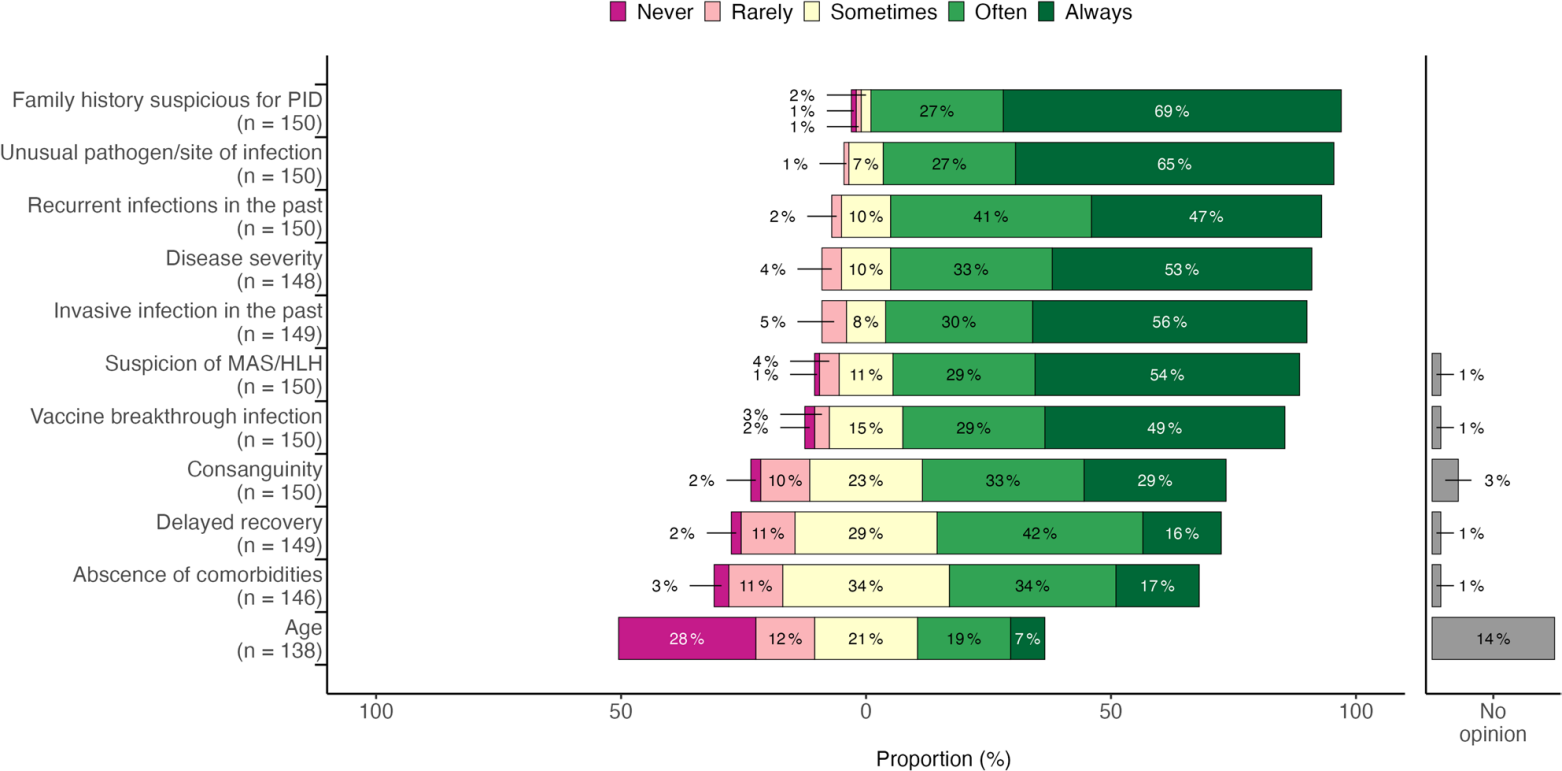
The graphs show which factors influence the respondents to almost always, often, sometimes, rarely, or never performing immunological testing in children presenting with community-acquired sepsis

Most respondents, 150/167 (90%), reported to be influenced by patient characteristics in their decision to perform immunological testing in paediatric patients after sepsis. A positive family history of immunodeficiency (96%), followed by an unusual site of infection or unusual pathogen causing sepsis (92%) were the most important factors prompting respondents almost always or often to perform follow-up investigations in children after sepsis, whereas age was reported to do so in only 26%. In terms of disease severity, fatal outcome and multi-organ failure almost always or often prompted respondents to order immunological testing in 70% and 60%, respectively (Fig. 3, Figure S1 and S2).

### Genetic investigations in children with sepsis

**Fig. 4:**
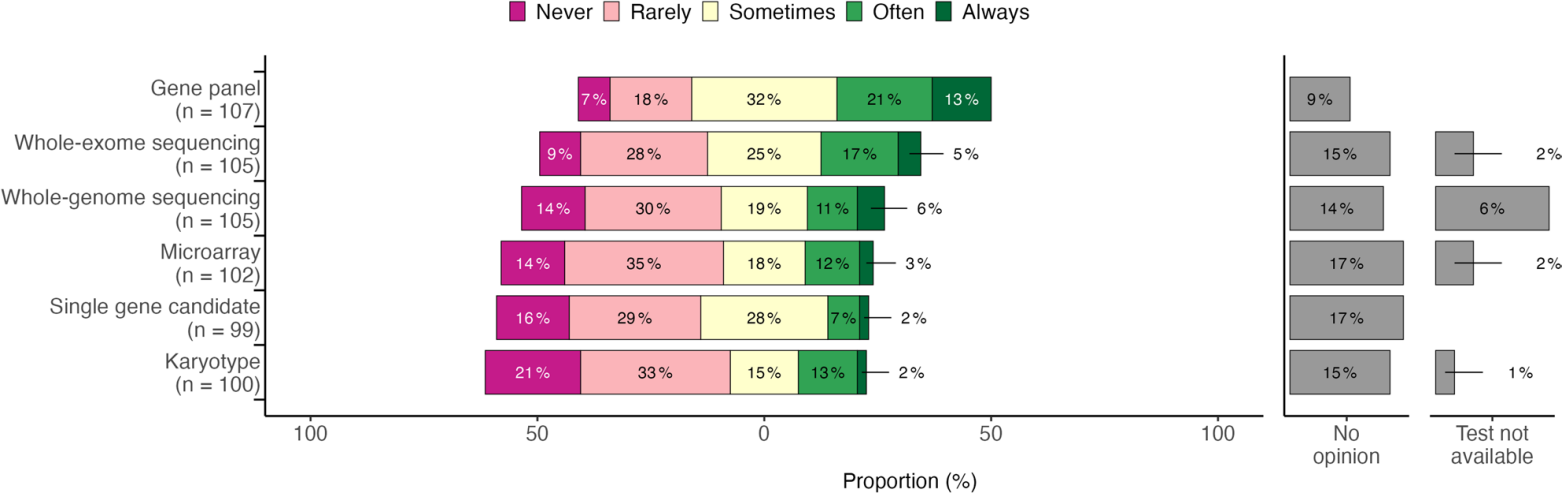
The graphs show if genetic testing is performed/ordered in children presenting with community-acquired sepsis, which are reported to be used almost always, often, sometimes, rarely, or never

Genetic testing was reported to be available on-site by 119/168 (71%) respondents, analysed in an external institution by 39/168 (23%), and 10/168 (6%) did not know. If genetic testing was reported to be performed, respondents stated to routinely use gene panels, followed by whole-exome sequencing (WES) (Fig. 4).

### Case vignettes

Response patterns are provided for each of the six case vignettes in the supplement (Figure S3-7). For scenario 2 (18-month-old girl with community-acquired *Pseudomonas aeruginosa* septic shock), scenario 4 (3-year-old boy with signs of septic shock who dies on ECMO without an identified pathogen), and scenario 6 (8-year-old girl with staphylococcal sepsis and necrotising pneumonia with an invasive infection in the past) the majority of respondents (90%, 80%, and 92%, respectively) opted to perform immunological work-up. Respondents stated to perform almost always or often total antibody levels in 85%, 92%, and 98%, in scenarios 2, 4, and 6. Lymphocyte subsets would be performed almost always or often by 79%, 85%, and 87% in scenarios 2, 4, and 6. For scenario 1 (4-year-old boy with bacteraemic but otherwise uncomplicated community acquired pneumococcal pneumonia), scenario 3 (6-year-old girl with bacteraemic meningococcal meninigitis, unvaccinated, and persistent neurocognitive dysfunctions), and scenario 5 (8-year-old girl with staphylococcal sepsis and necrotising pneumonia after *Influenza A* infection) approximately half of the respondents opted to perform immunological work-up with 57%, 64%, and 49%, respectively. Respondents stated to perform almost always or often total antibody levels in 93%, 92%, and 95%, in scenarios 1, 3, and 5; followed by 75% vaccine antibodies in scenario 1, 77% complement testing in scenario 3, and 70% lymphocyte subsets in scenario 5.

Genetic testing was answered to be performed by 50% of the respondents in scenario 4, 22% in scenario 6, and 18% in scenario 2. For the scenarios 1, 3, and 5, less than 7% answered to perform genetic testing. Additionally, a dihydrorhodamin (DHR) test and blood smear or abdominal ultrasound were occasionally requested as other tests.

## Discussion

This international survey of over 150 predominantly Europe-based paediatric specialists investigated diagnostic procedures in relation to immunological investigations in children after sepsis. Our results show that there is a wide range of self-reported practice and an absence of protocols or guidelines to inform best practice. Overall, particular patient characteristics were stated to impact specific decisions. A positive family history, an unusual site of infection, or an unusual pathogen responsible for sepsis were stated as the three main factors triggering immunological or genetic follow-up investigations. Also, fatal outcome or particularly severe course influenced physicians towards immunologically investigating children, whereas age seemed to be of less relevance. According to our data, genetic testing in the follow-up of paediatric sepsis patients, however, remains still rarely used. To our knowledge, this is the first survey investigating if and how healthcare professionals investigate children after sepsis. IEI at population level is rare, and is estimated at approximately 0.01% [16–18]. To improve the identification of patients with a higher risk of undiagnosed IEI, different tools to predict IEI were established, such as the “10 warning signs” of IEI and the immunodeficiency-related scores by Cunningham-Rundles et al. [19, 20]. However, the sensitivity of these scores varies between 33% and 64% in mixed populations, including adults and children, and some warning signs (e.g. ≥2 deep-seated infections including sepsis) are more important than others [21]. Overall, positive family history is often stated as the most significant single predictor of IEI [22, 23], however, most of these data stem from selected populations with a high proportion of consanguinity. Other known relevant risk factors for an underlying IEI are the need for intravenous antibiotics [22, 23], recurrent deep-seated infections, and a history of sibling death [22, 23]. A similar history- and risk-based approach seemed to be incorporated by the majority of respondents, who predominantly stated to perform immunological investigations in paediatric patients after sepsis if additional factors are present. In line with this, the factor stated as most important in impacting this decision was a family history of immunodeficiency. In the case scenarios, severity was another driver of the respondents’ decision to perform immunological investigations, whereas patient age mattered less.

We were interested in understanding which quantitative and functional tests were considered useful for identifying or ruling out possible IEI in children with sepsis. While some specific infections or pathogens are indicators for a specific type of immunodeficiency, such as complement defects in patients with recurrent Neisseria infection or granulocyte dysfunction in patients with recurrent abscesses, this is less clear in patients with community-acquired sepsis. In specific scenarios, targeted quantitative or functional immunological investigations might be efficient and effective [24, 25], however it remains unclear if such an approach is informative in broader groups of children with sepsis. Retrospective data from a Central European University Children’s Hospital including 360 previously healthy paediatric patients with community-acquired severe bacterial infection revealed a 9–18% rate of possibly actionable immune abnormalities detected by quantitative or functional testing at follow-up and the clinical picture of sepsis was associated with an increased likelihood of detecting such immune abnormalities [26]. In our survey, respondents often opted to perform total antibody levels, HIV serology, and lymphocyte subsets as first-tier tests, but only rarely ordered lymphocyte proliferation or lymphocyte phenotyping.

Over the last few years, increasing evidence for genetic variants in patients who experienced sepsis has become available and is considered to be more common than previously thought [12, 13, 24]. From our survey results, however, genetics have not yet been widely established in scenarios of children with sepsis. Around a quarter of respondents reported not having genetic testing on site and 59% reported that genetic testing was only available through a geneticist or a research project which might complicate the conduction of genetic testing. Lastly, turn-around time and high cost might further influence choices on genetic testing.

This survey-based study has several limitations. First, we were not able to retrieve the member count of each society and therefore, we were not able to report exact response rates. The predominance of Europe-based healthcare professionals may cause bias and the responses may be less representative of clinical practice in non-European settings. Second, the majority of respondents reported primarily working in Paediatric Infectious Diseases compared to only a small proportion of respondents working in Paediatric Intensive Care or Paediatric Immunology. While immunological testing is usually performed after an acute episode of sepsis, these follow-ups are rarely performed by PICU physicians but by immunologists or even more commonly Paediatric Infectious Disesase specialists or General Paediatricians. Therefore, our design may lead to an underrepresentation of other specialist, particularly General Paediatricians, and may not sufficiently capture their approach to post-PICU follow-up. Third, four out of five reported working at least partially in a tertiary centre. This overrepresentation of tertiary care medicine might both represent selection bias of the survey as well as reflect sites where children with sepsis are commonly cared for. Overall, the representativeness of the results cannot be fully established, however, we consider it noteworthy that a large proportion of responses originated from countries with high research and clinical activity in the field of paediatric sepsis. Therefore, while not fully representative, the responses likely reflect practices and perspectives from centres that are actively engaged in the field and will impact the field of sepsis in the future.

In conclusion, the results of this survey highlight the range of practices surrounding immunological investigations of children with sepsis. By capturing responses from a range of clinicians practicing internationally and presenting them structured clinical scenarios, the study findings highlight variation in practice. This can inform the development of a consensus-based algorithm for standardised immunological and genetic testing in children after sepsis, given the increased occurrence of IEI in children with sepsis.

## Electronic supplementary material

Below is the link to the electronic supplementary material.

Supplementary Material 1

## Data Availability

Data will be made available upon request directed to the corresponding author and approved by all authors on a case-by-case basis.
