# Supplementary material for: International survey on immunological diagnostics in children with sepsis
Source: Intensive Care Med Paediatr Neonatal. 2026 May 29;4(1):21. doi: 10.1007/s44253-026-00120-w (PMC13423901; doi:10.1007/s44253-026-00120-w)
Supplement: Supplementary file 1 — Supplementary Material 1 [file 44253_2026_120_MOESM1_ESM.pdf]

INTERNATIONAL SURVEY ON IMMUNOLOGICAL DIAGNOSTICS IN CHILDREN WITH SEPSIS

Nina Schöbi [1], Philipp Agyeman [1] , Vanessa Sancho-Shimizu [2], Johannes Trück [3], Julie Fitzgerald[4], Luregn J Schlapbach [5,6]

Content

Survey	Survey instrument
Letter	Cover letter to the societies
Table S1	Respondents characteristics
Figure S1	Factor - Severity
Figure S2	Factor - Age
Figure S3-S8	Case vignettes, scenarios 1-6

Paediatric Sepsis: A Survey Investigating Immunological Follow-Up in Children After Sepsis

Introduction

We are a group of paediatric infectious diseases specialists, immunologists, and intensivists interested in children with sepsis. Sepsis is a leading cause of death and disability in children, globally accounting for over one million childhood deaths per year. In high-income countries, 35 to 50% of paediatric sepsis deaths occur in previously healthy infants despite access to vaccination, health care, and effective antibiotics (Schlapbach et al., 2015; Weiss et al., 2015; Hartman et al., 2013; Launay et al., 2014). Traditionally, primary immunodeficiency is considered to be extremely rare at population level and, currently, there is no guidance to advise on immunologic or genetic investigations after a first sepsis episode (Asgari et al., 2016); Randolph and McCulloh, 2014; Borghesi, 2020).

With this survey, we seek to understand how immunologists, infectious diseases specialists, and paediatric intensivists immunologically investigate children with sepsis. Please be aware that once you have submitted your answers, you cannot go back.

Definition: With "sepsis" we refer to children with infection and associated organ dysfunction (this includes children with septic shock; and children with other infection-associated organ dysfunctions).

In advance, we thank you very much for participating and completing the attached questionnaire.

Responses cannot be traced back to you and are evaluated anonymously. Furthermore, the questionnaire was submitted to the local ethics committee; due to the set-up, this survey does not fall under the Human Research Act.

Consent

By filling out this survey, you consent to the contained data to be analysed and used for research purposes. The survey does not include identifiable data.

- ◯ I agree
- ◯ I do not agree

Paediatric Sepsis: A Survey Investigating Immunological Follow-Up in Children After Sepsis

Background Information

1. Are you treating children as a medical doctor or contribute to patient care decisions (through multidisciplinary team (MDT) meetings)?	◯ Yes<br>◯ No

2. Year of graduation from medical school:

◯ 1960
◯ 1961
◯ 1962
◯ 1963
◯ 1964
◯ 1965
◯ 1966
◯ 1967
◯ 1968
◯ 1969
◯ 1970
◯ 1971
◯ 1972
◯ 1973
◯ 1974
◯ 1975
◯ 1976
◯ 1977
◯ 1978
◯ 1979
◯ 1980
◯ 1981
◯ 1982
◯ 1983
◯ 1984
◯ 1985
◯ 1986
◯ 1987
◯ 1988
◯ 1989
◯ 1990
◯ 1991
◯ 1992
◯ 1993
◯ 1994
◯ 1995
◯ 1996
◯ 1997
◯ 1998
◯ 1999
◯ 2000
◯ 2001
◯ 2002
◯ 2003
◯ 2004
◯ 2005
◯ 2006
◯ 2007
◯ 2008
◯ 2009
◯ 2010
◯ 2011
◯ 2012
◯ 2013
◯ 2014
◯ 2015
◯ 2016
◯ 2017
◯ 2018
◯ 2019
◯ 2020
◯ 2021
◯ 2022
◯ 2023

3. Main discipline you are working in at present:

- ◯ Paediatric Infectious Diseases
- ◯ Paediatric Immunology
- ◯ Paediatric Infectious Diseases/Immunology (combined)
- ◯ Paediatric Intensive Care
- ◯ Paediatric Anesthesiology
- ◯ Adult Infectious Diseases
- ◯ Adult Immunology
- ◯ Adult Infectious Diseases/Immunology (combined)
- ◯ Adult Intensive Care
- ◯ Adult Anesthesiology
- ◯ General Paediatrician (hospital based)
- ◯ General Practice (private practice)
- ◯ Research only
- ◯ Other

If other discipline, please indicate: __________________________________

4. Your current role(s):

- ☐ Doctor during general Training/Residency
- ☐ Clinical Fellow
- ☐ Research Fellow
- ☐ Consultant/Attending Physician
- ☐ Other

(Please tick all that apply)

If other role, please indicate: __________________________________

5. Specify the type of centre(s) where you work:

- ☐ Tertiary/University Hospital
- ☐ Large/District Hospital
- ☐ Small/Community Hospital
- ☐ Private Practice
- ☐ Laboratory
- ☐ Research
- ☐ Industry/Pharma
- ☐ Other

(Please tick all that apply)

If other type, please indicate: __________________________________

6. Country in which you are practicing:

◯ Afghanistan
◯ Aland Islands
◯ Albania
◯ Algeria
◯ American Samoa
◯ Andorra
◯ Angola
◯ Anguilla
◯ Antarctica
◯ Antigua and Barbuda
◯ Argentina
◯ Armenia
◯ Aruba
◯ Australia
◯ Austria
◯ Azerbaijan
◯ Bahamas
◯ Bahrain
◯ Bangladesh
◯ Barbados
◯ Belarus
◯ Belgium
◯ Belize
◯ Benin
◯ Bermuda
◯ Bhutan
◯ Bolivia
◯ Bosnia and Herzegovina
◯ Botswana
◯ Bouvet Island
◯ Brazil
◯ British Virgin Islands
◯ British Indian Ocean Territory
◯ Brunei Darussalam
◯ Bulgaria
◯ Burkina Faso
◯ Burundi
◯ Cambodia
◯ Cameroon
◯ Canada
◯ Cape Verde
◯ Cayman Islands
◯ Central African Republic
◯ Chad
◯ Chile
◯ China
◯ Hong Kong, SAR China
◯ Macao, SAR China
◯ Christmas Island
◯ Cocos (Keeling) Islands
◯ Colombia
◯ Comoros
◯ Congo (Brazzaville)
◯ Congo, (Kinshasa)
◯ Cook Islands
◯ Costa Rica
◯ Côte d'Ivoire
◯ Croatia
◯ Cuba
◯ Cyprus
◯ Czech Republic
◯ Denmark
◯ Djibouti
◯ Dominica
◯ Dominican Republic
◯ Ecuador
◯ Egypt
◯ El Salvador
◯ Equatorial Guinea

◯ Eritrea
◯ Estonia
◯ Ethiopia
◯ Falkland Islands (Malvinas)
◯ Faroe Islands
◯ Fiji
◯ Finland
◯ France
◯ French Guiana
◯ French Polynesia
◯ French Southern Territories
◯ Gabon
◯ Gambia
◯ Georgia
◯ Germany
◯ Ghana
◯ Gibraltar
◯ Greece
◯ Greenland
◯ Grenada
◯ Guadeloupe
◯ Guam
◯ Guatemala
◯ Guernsey
◯ Guinea
◯ Guinea-Bissau
◯ Guyana
◯ Haiti
◯ Heard and Mcdonald Islands
◯ Holy See (Vatican City State)
◯ Honduras
◯ Hungary
◯ Iceland
◯ India
◯ Indonesia
◯ Iran, Islamic Republic of
◯ Iraq
◯ Ireland
◯ Isle of Man
◯ Israel
◯ Italy
◯ Jamaica
◯ Japan
◯ Jersey
◯ Jordan
◯ Kazakhstan
◯ Kenya
◯ Kiribati
◯ Korea (North)
◯ Korea (South)
◯ Kuwait
◯ Kyrgyzstan
◯ Lao PDR
◯ Latvia
◯ Lebanon
◯ Lesotho
◯ Liberia
◯ Libya
◯ Liechtenstein
◯ Lithuania
◯ Luxembourg
◯ Macedonia, Republic of
◯ Madagascar
◯ Malawi
◯ Malaysia
◯ Maldives
◯ Mali
◯ Malta
◯ Marshall Islands
◯ Martinique
◯ Mauritania

◯ Mauritius
◯ Mayotte
◯ Mexico
◯ Micronesia, Federated States of
◯ Moldova
◯ Monaco
◯ Mongolia
◯ Montenegro
◯ Montserrat
◯ Morocco
◯ Mozambique
◯ Myanmar
◯ Namibia
◯ Nauru
◯ Nepal
◯ Netherlands
◯ Netherlands Antilles
◯ New Caledonia
◯ New Zealand
◯ Nicaragua
◯ Niger
◯ Nigeria
◯ Niue
◯ Norfolk Island
◯ Northern Mariana Islands
◯ Norway
◯ Oman
◯ Pakistan
◯ Palau
◯ Palestinian Territory
◯ Panama
◯ Papua New Guinea
◯ Paraguay
◯ Peru
◯ Philippines
◯ Pitcairn
◯ Poland
◯ Portugal
◯ Puerto Rico
◯ Qatar
◯ Réunion
◯ Romania
◯ Russian Federation
◯ Rwanda
◯ Saint-Barthélemy
◯ Saint Helena
◯ Saint Kitts and Nevis
◯ Saint Lucia
◯ Saint-Martin (French part)
◯ Saint Pierre and Miquelon
◯ Saint Vincent and Grenadines
◯ Samoa
◯ San Marino
◯ Sao Tome and Principe
◯ Saudi Arabia
◯ Senegal
◯ Serbia
◯ Seychelles
◯ Sierra Leone
◯ Singapore
◯ Slovakia
◯ Slovenia
◯ Solomon Islands
◯ Somalia
◯ South Africa
◯ South Georgia and the South Sandwich Islands
◯ South Sudan
◯ Spain
◯ Sri Lanka
◯ Sudan
◯ Suriname

◯ Svalbard and Jan Mayen Islands
◯ Swaziland
◯ Sweden
◯ Switzerland
◯ Syrian Arab Republic (Syria)
◯ Taiwan, Republic of China
◯ Tajikistan
◯ Tanzania, United Republic of
◯ Thailand
◯ Timor-Leste
◯ Togo
◯ Tokelau
◯ Tonga
◯ Trinidad and Tobago
◯ Tunisia
◯ Turkey
◯ Turkmenistan
◯ Turks and Caicos Islands
◯ Tuvalu
◯ Uganda
◯ Ukraine
◯ United Arab Emirates
◯ United Kingdom
◯ United States of America
◯ US Minor Outlying Islands
◯ Uruguay
◯ Uzbekistan
◯ Vanuatu
◯ Venezuela (Bolivarian Republic)
◯ Viet Nam
◯ Virgin Islands, US
◯ Wallis and Futuna Islands
◯ Western Sahara
◯ Yemen
◯ Zambia
◯ Zimbabwe

---

7. Is there a dedicated Paediatric Immunology service in your centre?

◯ Yes
◯ No

---

8. In your centre, immunological tests are generally ordered by:

☐ Paediatric Immunology
☐ Paediatric Infectious Diseases
☐ Any treating medical team member
☐ Other

If other agent, please indicate:

____

---

9. How many times per year do you order immunological tests by yourself (including, but not limited to, total antibody levels, specific antibodies (e.g. against vaccine antigens), lymphocyte subsets etc.)?

◯ Never
◯ 1-10 times
◯ 11-50 times
◯ >50 times

Paediatric Sepsis: A Survey Investigating Immunological Follow-Up in Children After Sepsis

General Questions

10. Which of the following tests do you routinely perform in children (>1 month to 18 years of age) presenting with community-acquired sepsis in the absence of known major comorbidities (e.g. iatrogenic immunosuppression, central venous access, chromosomal syndromes, major chronic handicap)?

	almost always	often	sometimes	rarely	never	I don't know
Total antibody levels (IgA, IgM, IgG) testing	○	○	○	○	○	○
IgG subclasses testing (on a first encounter)	○	○	○	○	○	○
Vaccine antibody testing	○	○	○	○	○	○
Lymphocyte subsets (T-cells, B-cells, NK-cells) testing	○	○	○	○	○	○
Extended lymphocyte phenotyping (T-cell precursors, B-cell subsets)	○	○	○	○	○	○
Lymphocyte proliferation testing	○	○	○	○	○	○
Complement testing: Alternative Pathway activity	○	○	○	○	○	○
Complement testing: Classical Pathway activity	○	○	○	○	○	○
Complement testing: C3, C4 concentrations	○	○	○	○	○	○
HIV serology testing	○	○	○	○	○	○
Other test? ______	○	○	○	○	○	○
Other test? ______	○	○	○	○	○	○
Other test? ______	○	○	○	○	○	○

11. Are there any patient characteristics that influence your decision to carry out immunological testing (e.g. unusual severity after presentation, patient history, age)?

○ Yes
○ No, I test all children with sepsis (irrespective of other factors)
(If not, please continue with Q14 below)

12. Please state whether the presentation of higher severity (in form of the following events) affect your decision to perform an immunological work-up:

	almost always	often	sometimes	rarely	never	I don't know
Death	○	○	○	○	○	○
PICU admission	○	○	○	○	○	○
Multi organ failure	○	○	○	○	○	○

Single organ failure (i.e. sepsis defined as infection with organ dysfunction)	◯	◯	◯	◯	◯	◯
Limb loss	◯	◯	◯	◯	◯	◯
Other events? ______	◯	◯	◯	◯	◯	◯

13. Please state whether the following factors influence your decision to carry out immunological testing:

	almost always	often	sometimes	rarely	never	I don't know
Unusual pathogen or unusual site of infection	◯	◯	◯	◯	◯	◯
Vaccine breakthrough infection (vaccine serotype / -group) in fully vaccinated child)	◯	◯	◯	◯	◯	◯
Unusual severity of presentation	◯	◯	◯	◯	◯	◯
Suspicion of immune activation syndrome (MAS/HLH)	◯	◯	◯	◯	◯	◯
Delayed recovery	◯	◯	◯	◯	◯	◯
Personal history positive for an invasive infection in the past	◯	◯	◯	◯	◯	◯
Recurrent infections	◯	◯	◯	◯	◯	◯
Family history (recurrent or severe/fatal infections, known immunodeficiency etc.)	◯	◯	◯	◯	◯	◯
Consanguinity	◯	◯	◯	◯	◯	◯
Absence of comorbidities that increase the risk of sepsis (e.g. neonatal age, prematurity, major chronic medical comorbidity, iatrogenic immunosuppression, hospitalisation/central venous access as risk factors)	◯	◯	◯	◯	◯	◯
Age groups (if yes, you will be asked to specify)	◯	◯	◯	◯	◯	◯
preterm/neonates	◯	◯	◯	◯	◯	◯
28 days - 2 years	◯	◯	◯	◯	◯	◯
2-5 years	◯	◯	◯	◯	◯	◯
5-12 years	◯	◯	◯	◯	◯	◯
>12 years	◯	◯	◯	◯	◯	◯
Other factors? ______	◯	◯	◯	◯	◯	◯

14. Does your centre use a documented guideline/algorithm for a standardised immunological work-up in children after sepsis?

◯ Yes, sepsis specific
◯ Yes, but not sepsis specific
◯ No

15. Do you have the possibility to order genetic testing at your institution?

◯ Yes
◯ No
◯ I don't know
(If not or you don't know, please continue with Q18 (next page))

16. If you perform/order genetic testing in children after sepsis, which of the following are commonly used by yourself?

	almost always	often	sometimes	rarely	never	not available	I don't know
Single gene candidate testing	◯	◯	◯	◯	◯	◯	◯
Microarray	◯	◯	◯	◯	◯	◯	◯
Karyotype	◯	◯	◯	◯	◯	◯	◯
Gene panel	◯	◯	◯	◯	◯	◯	◯
Whole-exome sequencing	◯	◯	◯	◯	◯	◯	◯
Whole-genome sequencing	◯	◯	◯	◯	◯	◯	◯

17. If you perform whole-exome or whole-genome sequencing, through which route(s) is it available?

☐ Research projects
☐ Clinician-directed genetic testing
☐ Geneticist-directed testing
(Please tick all that apply)

Paediatric Sepsis: A Survey Investigating Immunological Follow-Up in Children After Sepsis

Case Scenarios

Scenario 1: A previously healthy 4-year-old boy presents with lobar pneumonia with growth of pneumococci in blood cultures, he does not need PICU support and recovers fully. Serotyping reveals a pneumococcus serotype 19. He is up-to-date with his immunisations (including PCV-13) and this is the first episode of an invasive infection. The family history is uneventful.

For this or similar scenarios:

18. Do you perform any immunological work-up?

◯ Yes
◯ No
(If not, please continue with Q20 below)

19. If you perform immunological testing, which of the following tests do you carry out?

	almost always	often	sometimes	rarely	never	not available	I don't know
Total antibody levels (IgA, IgM, IgG) testing	◯	◯	◯	◯	◯	◯	◯
IgG subclasses testing (on a first encounter)	◯	◯	◯	◯	◯	◯	◯
Vaccine antibody testing	◯	◯	◯	◯	◯	◯	◯
Lymphocyte subsets (T-, B-, and NK-cells) testing	◯	◯	◯	◯	◯	◯	◯
Lymphocyte proliferation testing	◯	◯	◯	◯	◯	◯	◯
Complement testing	◯	◯	◯	◯	◯	◯	◯
HIV serology testing	◯	◯	◯	◯	◯	◯	◯

If you perform vaccine antibody testing, please specify the context:

◯ Generally
◯ Only if vaccine failure

If you perform complement testing, please specify:

☐ Alternative complement pathway activity (AP50)
☐ Classical complement pathway activity (CH10)
☐ Complement C3/C4 concentrations
(Please tick all that apply)

If you perform HIV testing, please indicate in which of the following cases:

☐ If the mother did not have an HIV test during pregnancy
☐ If the mother is known to be HIV positive
☐ If the patient was born in a country with high HIV prevalence
☐ regardless of the mother's HIV status or local prevalence
(Please tick all that apply)

20. Do you perform genetic testing?

◯ Yes
◯ Only if abnormal immunological testing
◯ Only if NO abnormal immunological testing
◯ No

21. Do you order any other work-up?

____

Paediatric Sepsis: A Survey Investigating Immunological Follow-Up in Children After Sepsis

Case Scenarios

Scenario 2: A previously healthy 18-month-old girl presents with community-acquired Pseudomonas aeruginosa septic shock. She is treated in PICU with ventilation and inotropes, and recovers completely. She is fully vaccinated and her family history is unremarkable.

For this or similar scenarios:

22. Do you perform any immunological work-up?

- ◯ Yes
- ◯ No

(If not, please continue with Q24 below)

23. If you perform immunological testing, which of the following tests do you carry out?

	almost always	often	sometimes	rarely	never	not available	I don't know
Total antibody levels (IgA, IgM, IgG) testing	◯	◯	◯	◯	◯	◯	◯
IgG subclasses testing (on a first encounter)	◯	◯	◯	◯	◯	◯	◯
Vaccine antibody testing	◯	◯	◯	◯	◯	◯	◯
Lymphocyte subsets (T-, B-, and NK-cells) testing	◯	◯	◯	◯	◯	◯	◯
Lymphocyte proliferation testing	◯	◯	◯	◯	◯	◯	◯
Complement testing	◯	◯	◯	◯	◯	◯	◯
HIV serology testing	◯	◯	◯	◯	◯	◯	◯

If you perform vaccine antibody testing, please specify the context:

- ◯ Generally
- ◯ Only if vaccine failure

If you perform HIV testing, please indicate in which of the following cases:

- ☐ If the mother did not have an HIV test during pregnancy
- ☐ If the mother is known to be HIV positive
- ☐ If the patient was born in a country with high HIV prevalence
- ☐ regardless of the mother's HIV status or local prevalence

(Please tick all that apply)

If you perform complement testing, please specify:

- ☐ Alternative complement pathway activity (AP50)
- ☐ Classical complement pathway activity (CH10)
- ☐ Complement C3/C4 concentrations

(Please tick all that apply)

24. Do you perform genetic testing?

- ◯ Yes
- ◯ Only if abnormal immunological testing
- ◯ Only if NO abnormal immunological testing
- ◯ No

25. Do you order any other work-up?

____

Paediatric Sepsis: A Survey Investigating Immunological Follow-Up in Children After Sepsis

Case Scenarios

Scenario 3: A 6-year-old girl presents with bacteraemic meningococcal meningitis serotype B. She is admitted to PICU, needs respiratory support and vasoactive drugs because of septic shock with multi-organ failure. She recovers slowly with neurocognitive dysfunctions remaining. She is up-to-date with her immunisations (including MenACWY but not MenB vaccination). This is the first episode of an invasive infection. The family history is uneventful.

For this or similar scenarios:

26. Do you perform any immunological work-up?

◯ Yes
◯ No
(If not, please continue with Q28 below)

27. If you perform immunological testing, which of the following tests do you carry out?

	almost always	often	sometimes	rarely	never	not available	I don't know
Total antibody levels (IgA, IgM, IgG) testing	◯	◯	◯	◯	◯	◯	◯
IgG subclasses testing (on a first encounter)	◯	◯	◯	◯	◯	◯	◯
Vaccine antibody testing	◯	◯	◯	◯	◯	◯	◯
Lymphocyte subsets (T-, B-, and NK-cells) testing	◯	◯	◯	◯	◯	◯	◯
Lymphocyte proliferation testing	◯	◯	◯	◯	◯	◯	◯
Complement testing	◯	◯	◯	◯	◯	◯	◯
HIV serology testing	◯	◯	◯	◯	◯	◯	◯

If you perform vaccine antibody testing, please specify the context:

◯ Generally
◯ Only if vaccine failure

If you perform complement testing, please specify:

☐ Alternative complement pathway activity (AP50)
☐ Classical complement pathway activity (CH10)
☐ Complement C3/C4 concentrations
(Please tick all that apply)

If you perform HIV testing, please indicate in which of the following cases:

☐ If the mother did not have an HIV test during pregnancy
☐ If the mother is known to be HIV positive
☐ If the patient was born in a country with high HIV prevalence
☐ regardless of the mother's HIV status or local prevalence
(Please tick all that apply)

28. Do you perform genetic testing?

◯ Yes
◯ Only if abnormal immunological testing
◯ Only if NO abnormal immunological testing
◯ No

29. Do you order any other work-up?

____

Paediatric Sepsis: A Survey Investigating Immunological Follow-Up in Children After Sepsis

Case Scenarios

Scenario 4: A previously healthy 3-year-old boy presents with septic shock after a history of a few days of pyrexia with respiratory symptoms. C-reactive protein is 354 mg/l and leukocytes are 1.8 G/l on presentation. Despite treatment with ECMO for refractory shock the child dies. Microbiological investigations intra vitam and post mortem result negative. The family history is uneventful.

For this or similar scenarios:

30. Do you perform any immunological work-up?

◯ Yes
◯ No
(If not, please continue with Q32 below)

31. If you perform immunological testing, which of the following tests do you carry out?

	almost always	often	sometimes	rarely	never	not available	I don't know
Total antibody levels (IgA, IgM, IgG) testing	◯	◯	◯	◯	◯	◯	◯
IgG subclasses testing (on a first encounter)	◯	◯	◯	◯	◯	◯	◯
Vaccine antibody testing	◯	◯	◯	◯	◯	◯	◯
Lymphocyte subsets (T-, B-, and NK-cells) testing	◯	◯	◯	◯	◯	◯	◯
Lymphocyte proliferation testing	◯	◯	◯	◯	◯	◯	◯
Complement testing	◯	◯	◯	◯	◯	◯	◯
HIV serology testing	◯	◯	◯	◯	◯	◯	◯

If you perform vaccine antibody testing, please specify the context:

◯ Generally
◯ Only if vaccine failure

If you perform complement testing, please specify:

☐ Alternative complement pathway activity (AP50)
☐ Classical complement pathway activity (CH10)
☐ Complement C3/C4 concentrations
(Please tick all that apply)

If you perform HIV testing, please indicate in which of the following cases:

☐ If the mother did not have an HIV test during pregnancy
☐ If the mother is known to be HIV positive
☐ If the patient was born in a country with high HIV prevalence
☐ regardless of the mother's HIV status or local prevalence
(Please tick all that apply)

32. Do you perform genetic testing?

◯ Yes
◯ Only if abnormal immunological testing
◯ Only if NO abnormal immunological testing
◯ No

33. Do you order any other work-up?

____

Paediatric Sepsis: A Survey Investigating Immunological Follow-Up in Children After Sepsis

Case Scenarios

Scenario 5: An 8-year-old previously healthy girl presents with staphylococcal sepsis and necrotizing pneumonia. She is positive for Influenza A. She requires inotropes and ventilation but eventually recovers. She is up-to-date with her immunisations. This is the first episode of an invasive infection. The family history is uneventful.

For this or similar scenarios:

34. Do you perform any immunological work-up?

◯ Yes
◯ No
(If not, please continue with Q36 below)

35. If you perform immunological testing, which of the following tests do you carry out?

	almost always	often	sometimes	rarely	never	not available	I don't know
Total antibody levels (IgA, IgM, IgG) testing	◯	◯	◯	◯	◯	◯	◯
IgG subclasses testing (on a first encounter)	◯	◯	◯	◯	◯	◯	◯
Vaccine antibody testing	◯	◯	◯	◯	◯	◯	◯
Lymphocyte subsets (T-, B-, and NK-cells) testing	◯	◯	◯	◯	◯	◯	◯
Lymphocyte proliferation testing	◯	◯	◯	◯	◯	◯	◯
Complement testing	◯	◯	◯	◯	◯	◯	◯
HIV serology testing	◯	◯	◯	◯	◯	◯	◯

If you perform vaccine antibody testing, please specify the context:

◯ Generally
◯ Only if vaccine failure

If you perform complement testing, please specify:

☐ Alternative complement pathway activity (AP50)
☐ Classical complement pathway activity (CH10)
☐ Complement C3/C4 concentrations
(Please tick all that apply)

If you perform HIV testing, please indicate in which of the following cases:

☐ If the mother did not have an HIV test during pregnancy
☐ If the mother is known to be HIV positive
☐ If the patient was born in a country with high HIV prevalence
☐ regardless of the mother's HIV status or local prevalence
(Please tick all that apply)

36. Do you perform genetic testing?

◯ Yes
◯ Only if abnormal immunological testing
◯ Only if NO abnormal immunological testing
◯ No

37. Do you order any other work-up?

____

Paediatric Sepsis: A Survey Investigating Immunological Follow-Up in Children After Sepsis

Case Scenarios

Scenario 6: An 8-year-old girl presents with staphylococcal sepsis and necrotizing pneumonia. She requires inotropes and ventilation but eventually recovers. She is up-to-date with her vaccines. This is the second episode of an invasive infection. At the age of 3 years she had presented with E. coli bacteraemia. The family history is uneventful.

For this or similar scenarios:

38. Do you perform any immunological work-up?

◯ Yes
◯ No
(If not, please continue with Q40 below)

39. If you perform immunological testing, which of the following tests do you carry out?

	almost always	often	sometimes	rarely	never	not available	I don't know
Total antibody levels (IgA, IgM, IgG) testing	◯	◯	◯	◯	◯	◯	◯
IgG subclasses testing (on a first encounter)	◯	◯	◯	◯	◯	◯	◯
Vaccine antibody testing	◯	◯	◯	◯	◯	◯	◯
Lymphocyte subsets (T-, B-, and NK-cells) testing	◯	◯	◯	◯	◯	◯	◯
Lymphocyte proliferation testing	◯	◯	◯	◯	◯	◯	◯
Complement testing	◯	◯	◯	◯	◯	◯	◯
HIV serology testing	◯	◯	◯	◯	◯	◯	◯

If you perform vaccine antibody testing, please specify the context:

◯ Generally
◯ Only if vaccine failure

If you perform complement testing, please specify:

☐ Alternative complement pathway activity (AP50)
☐ Classical complement pathway activity (CH10)
☐ Complement C3/C4 concentrations
(Please tick all that apply)

If you perform HIV testing, please indicate in which of the following cases:

☐ If the mother did not have an HIV test during pregnancy
☐ If the mother is known to be HIV positive
☐ If the patient was born in a country with high HIV prevalence
☐ regardless of the mother's HIV status or local prevalence
(Please tick all that apply)

40. Do you perform genetic testing?

◯ Yes
◯ Only if abnormal immunological testing
◯ Only if NO abnormal immunological testing
◯ No

41. Do you order any other work-up?

____

Paediatric Sepsis: A Survey Investigating Immunological Follow-Up in Children After Sepsis

Perceived Utility and Service Delivery

42. Do you think an algorithm for a standardised immunological work-up for children after sepsis would be helpful?	◯ Yes<br>◯ No<br>◯ I don't know

43. If such a guideline existed, how important would endorsement be by your:

	almost always	often	sometimes	rarely	never	I don't know
National specialist association	◯	◯	◯	◯	◯	◯
International specialist association	◯	◯	◯	◯	◯	◯
Sepsis campaign	◯	◯	◯	◯	◯	◯

Dear Colleagues,

We are a group of paediatric infectious diseases specialists, immunologists, and intensivists interested in children with sepsis. Sepsis is a leading cause of death and disability in children, globally accounting for over one million childhood deaths per year. In high-income countries, 35 to 50% of paediatric sepsis deaths occur in previously healthy infants despite access to vaccination, health care, and effective antibiotics (Schlapbach et al., 2015; Weiss et al., 2015; Hartman et al., 2013; Launay et al., 2014). Traditionally, primary immunodeficiency is considered to be extremely rare at population level and, currently, there is no guidance to advise on immunologic or genetic investigations after a first sepsis episode (Asgari et al., 2016); Randolph and McCulloh, 2014; Borghesi, 2020).

With this survey, we seek to understand how immunologists, infectious diseases specialists, and paediatric intensivists immunologically investigate children with sepsis.

This survey is designed on RedCap and counts 44 questions. Completing these questions should take approximately 15 minutes.

The questionnaire was submitted to the ethics committee in Switzerland; due to the set-up, this survey does not fall under the Human Research Act. Responses cannot be traced back to respondents.

https://kispiredcap.uzh.ch/redcap/surveys/?s=L8RMLC4NXNN7P9LR

Kind regards,

Nina Schöbi

Nina Schöbi (Consultant in Paediatric Infectious Disease, Bern, Switzerland)

Philipp Agyeman (Consultant in Paediatric Infectious Disease, Bern, Switzerland)

Johannes Trück (Consultant in Paediatric Immunology, Zurich, Switzerland)

Vanessa Sancho-Shimizu (Research Scientist, Imperial College London, England)

Luregn Schlapbach (Head of Paediatric Intensive Care, Zurich, Switzerland)

Table S1: Respondents characteristics

	n (%)
**Work experience**	
0-10 years	30 (18%)
11-20 years	72 (43%)
20 years	66 (39%)
**Job position**	
Assistant doctor	18 (11%)
Fellow	16 (10%)
Consultant	127 (76%)
Researcher	4 (2%)
Other	2 (1%)
Not answered	1 (1%)
**Job role**	
Paediatric Infectious Diseases	89 (53%)
General Paediatrician	31 (18%)
Paediatric Immunologist	19 (11%)
Paediatric Intensivist	9 (5%)
General Practitioner	2 (1%)
Oncology	2 (1%)
Researcher	2 (1%)
Other	3 (2%)
Not answered	11 (7%)
**Workplace**	
Private Practice	4 (2%)
Community Hospital	6 (4%)
District Hospital	23 (14%)
Tertiary Hospital	135 (80%)
**Decisions on diagnostics taken by**	
Immunologist	79 (47%)
Infectious Diseases Specialist	41 (24%)
Medical Team	46 (27%)
Not answered	2 (1%)
**How many times do you order immunological follow-up per year**	
Never	8 (5%)
Less than 10 times	53 (32%)
Between 11 and 50 times	73 (43%)
More than 50 times	34 (20%)

Figure S1: Factor - Severity

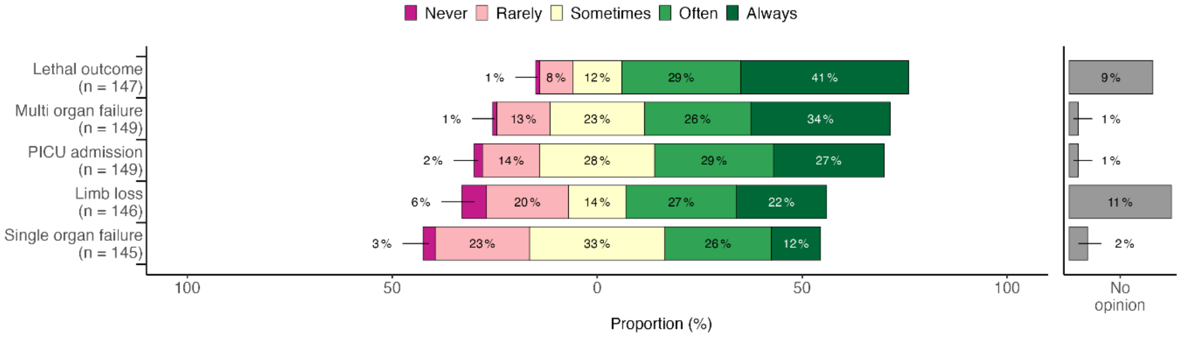

The graphs show how disease severity influences the respondents to almost always, often, sometimes, rarely, or never performing immunological testing in children presenting with community-acquired sepsis.

Figure S2: Factor – Age

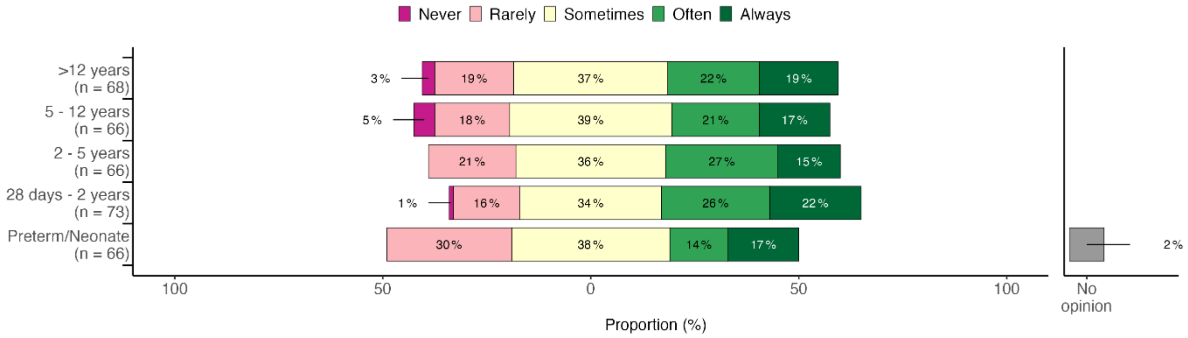

The graphs show how age influences the respondents to almost always, often, sometimes, rarely, or never performing immunological testing in children presenting with community-acquired sepsis.

Figure S3-S8: Case vignettes, scenarios 1-6

Case vignette

A previously healthy 4-year-old boy presents with lobar pneumonia with growth of pneumococci in blood cultures. He does not need intensive care support and recovers fully. Serotyping reveals a pneumococcus serotype 19. He is up-to-date with his immunisations (including PCV-13) and this is the first episode of an invasive infection. The family history is uneventful.

57% n = 164

Percentage performing immunological work-up

Immunological testing respondents would perform

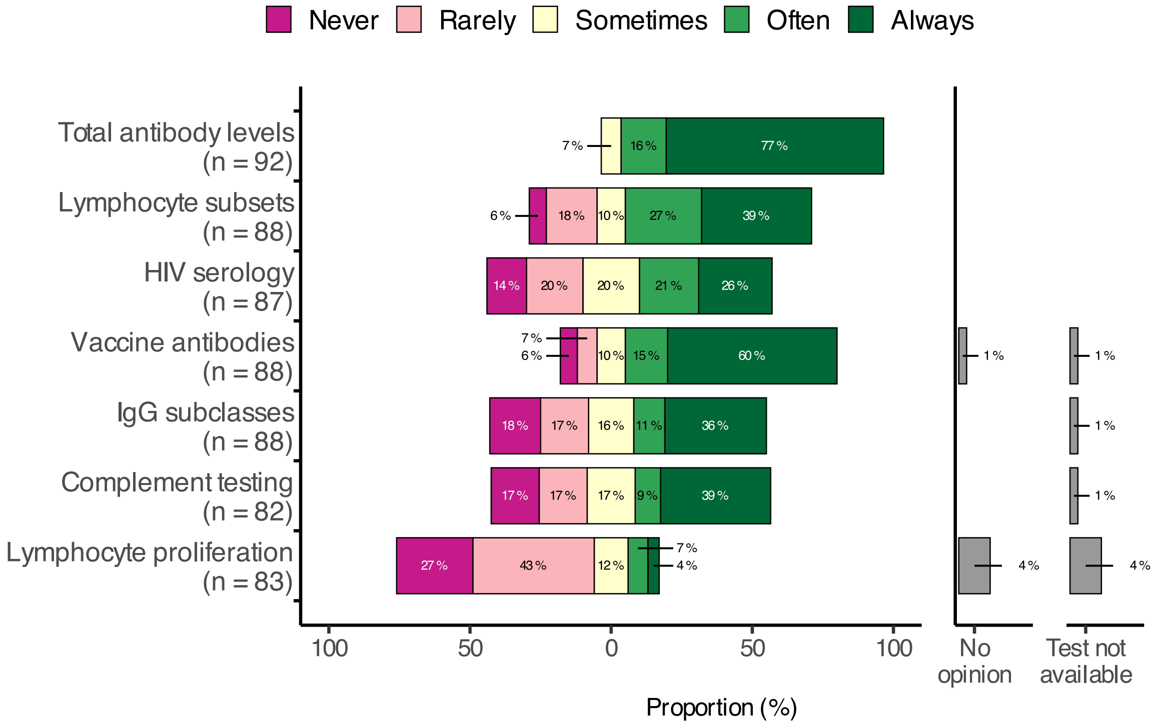

Details on immunological tests

When to test vaccine antibodies	Vaccine failure **44%** n = 81	Always **56%** n = 81		
Complement test to perform	C3/C4 **63%** n = 67	AP50 **55%** n = 67	CH100 **60%** n = 67	
When to perform HIV test	Mum HIV infected **36%** n = 75	No test in pregnancy **35%** n = 75	High-prev country **44%** n = 75	Always **49%** n = 75
Would you do genetic testing?	No **65%** n = 161	Abnormal immunology **25%** n = 161	Normal immunology **5%** n = 161	Yes **5%** n = 161

Additional tests

In addition, 9 respondents considered an abdominal ultrasound to confirm the presence of a spleen and 4 a blood smear to exclude Howell-Jolly bodies important. One respondent each asked for mannose binding lectin, dihydrorhodamin test, and TLR-NF-kappa B errors.

Scenario 1

Case vignette

A previously healthy 18-month-old girl presents with community-acquired Pseudomonas aeruginosa septic shock. She is treated in intensive care with ventilation and inotropes, and recovers completely. She is fully vaccinated and her family history is unremarkable.

90% n = 155

Percentage performing immunological work-up

Immunological testing respondents would perform

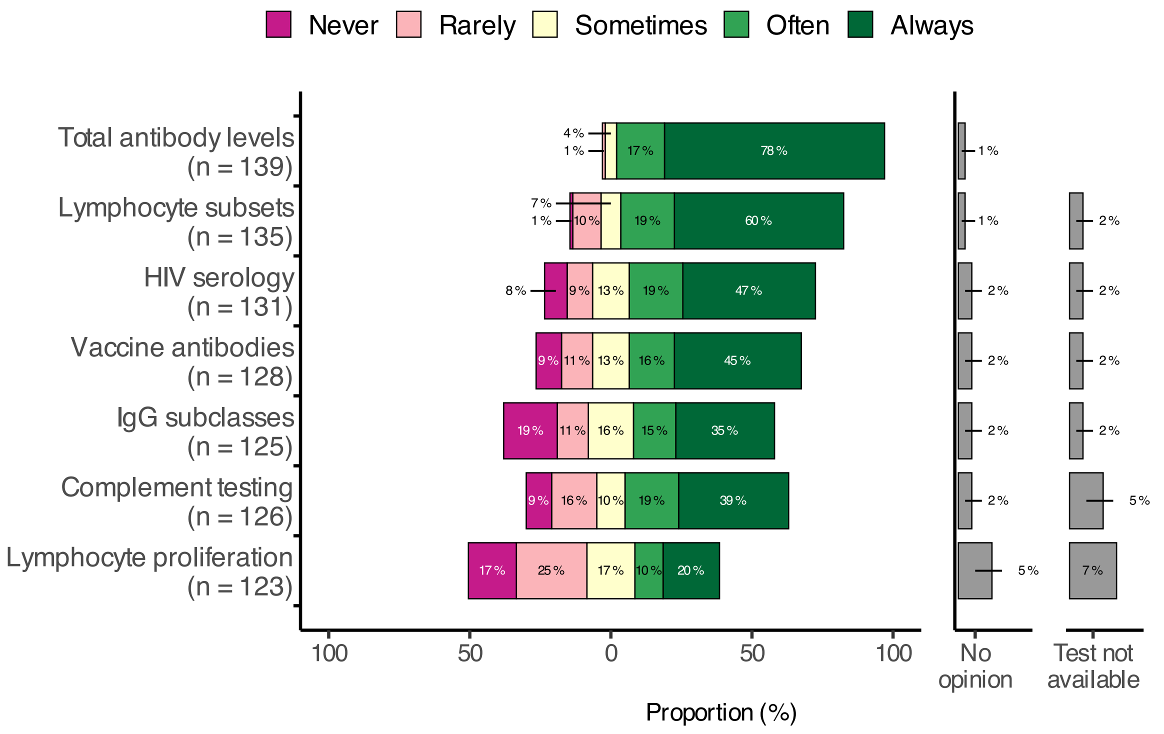

Details on immunological tests

When to test vaccine antibodies		Vaccine failure **21%** n = 110	Always **76%** n = 110	
Complement test to perform		C3/C4 **72%** n = 106	AP50 **51%** n = 106	CH100 **50%** n = 106
When to perform HIV test	Mum HIV infected **31%** n = 115	No test in pregnancy **24%** n = 115	High-prev country **32%** n = 115	Always **59%** n = 115
Would you do genetic testing?	No **35%** n = 155	Abnormal immunology **40%** n = 155	Normal immunology **7%** n = 155	Yes **18%** n = 155

Additional tests

In addition, 8 respondents requested a dihydrorhodamin test to exclude chronic granulomatous disease. Three respondents recommended to exclude cystic fibrosis. Lastly, one respondent each asked for a full blood count and mannose binding lectin.

Scenario 2

Case vignette

A 6-year-old girl presents with bacteraemic meningococcal meningitis serotype B. She is admitted to intensive care, needs respiratory support and vasoactive drugs because of septic shock with multi-organ failure. She recovers slowly with neurocognitive dysfunctions remaining. She is up-to-date with her immunisations (including MenACWY but not MenB vaccination). This is the first episode of an invasive infection. The family history is uneventful.

64%

n = 154

Percentage performing immunological work-up

Immunological testing respondents would perform

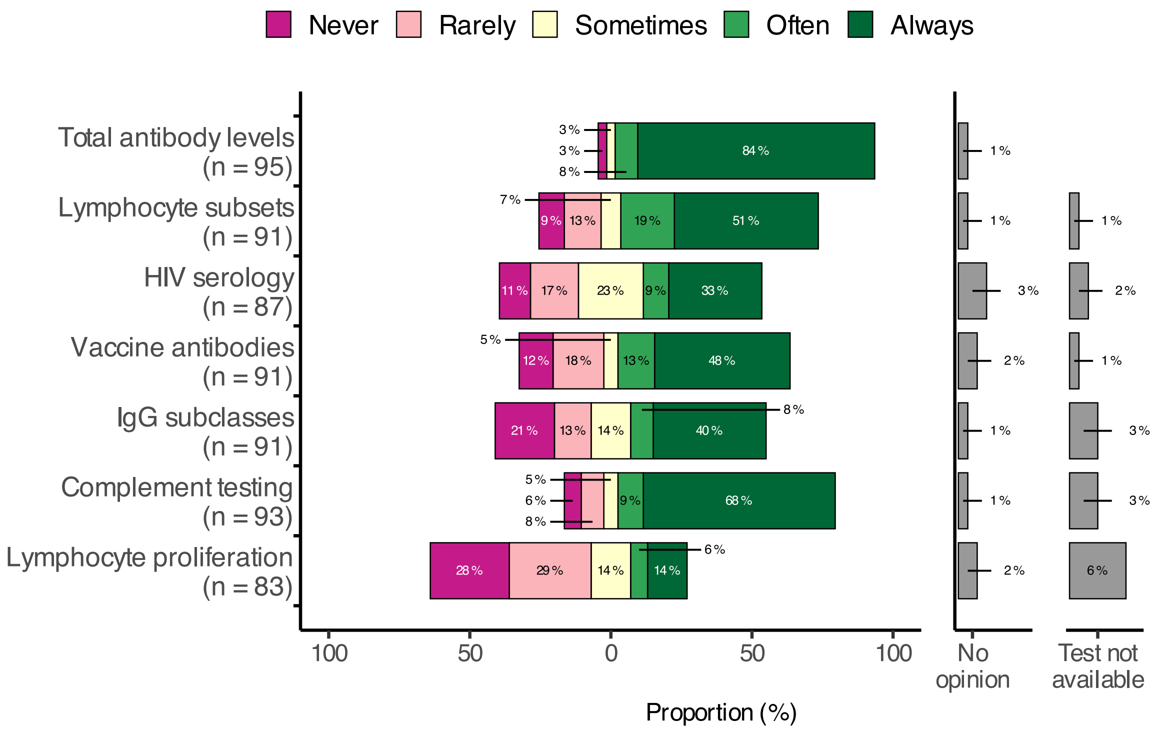

Details on immunological tests

When to test vaccine antibodies		Vaccine failure<br>**27%**<br>n = 77	Always<br>**70%**<br>n = 77	
Complement test to perform		C3/C4<br>**65%**<br>n = 83	AP50<br>**66%**<br>n = 83	CH100<br>**66%**<br>n = 83
When to perform HIV test	Mum HIV infected<br>**32%**<br>n = 72	No test in pregnancy<br>**29%**<br>n = 72	High-prev country<br>**32%**<br>n = 72	Always<br>**60%**<br>n = 72
Would you do genetic testing?	No<br>**54%**<br>n = 152	Abnormal immunology<br>**36%**<br>n = 152	Normal immunology<br>**5%**<br>n = 152	Yes<br>**6%**<br>n = 152

Additional tests

In addition, 5 respondents requested an abdominal ultrasound to confirm the presence of a spleen and 2 a blood smear to exclude Howell-Jolly bodies. One respondent asked for mannose binding lectin.

Scenario 3

Case vignette

A previously healthy 3-year-old boy presents with septic shock after a history of a few days of pyrexia with respiratory symptoms. C-reactive protein is 354 mg/l and leukocytes are 1.8 G/l on presentation. Despite treatment with ECMO for refractory shock the child dies. Microbiological investigations intra vitam and post mortem result negative. The family history is uneventful.

80% Percentage performing immunological work-up

n = 152

Immunological testing respondents would perform

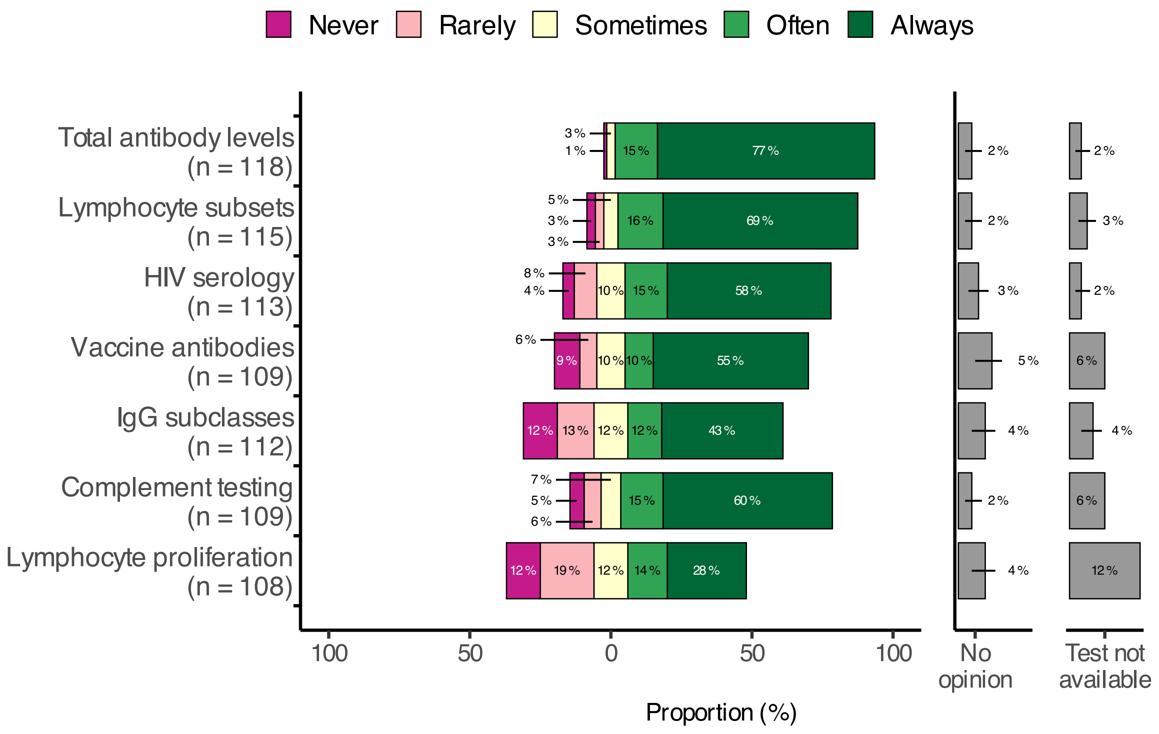

Details on immunological tests

When to test vaccine antibodies	Vaccine failure **18%** n = 88	Always **80%** n = 88		
Complement test to perform	C3/C4 **72%** n = 96	AP50 **61%** n = 96	CH100 **66%** n = 96	
When to perform HIV test	Mum HIV infected **28%** n = 103	No test in pregnancy **20%** n = 103	High-prev country **26%** n = 103	Always **67%** n = 103
Would you do genetic testing?	No **30%** n = 147	Abnormal immunology **16%** n = 147	Normal immunology **5%** n = 147	Yes **50%** n = 147

Additional tests

In addition, 3 respondents requested investigations around the diagnosis of haemophagocytic lymphohistiocytosis, one respondent each asked for an abdominal ultrasound to confirm the presence of a spleen, a full blood count, mannose binding lectin, and dihydrorhodamin test. Furthermore, skin biopsy and autopsy were mentioned.

Scenario 4

Case vignette

An 8-year-old previously healthy girl presents with staphylococcal sepsis and necrotizing pneumonia. She is positive for Influenza A. She requires inotropes and ventilation but eventually recovers. She is up-to-date with her immunisations. This is the first episode of an invasive infection. The family history is uneventful.

49%

n = 151

Percentage performing immunological work-up

Immunological testing respondents would perform

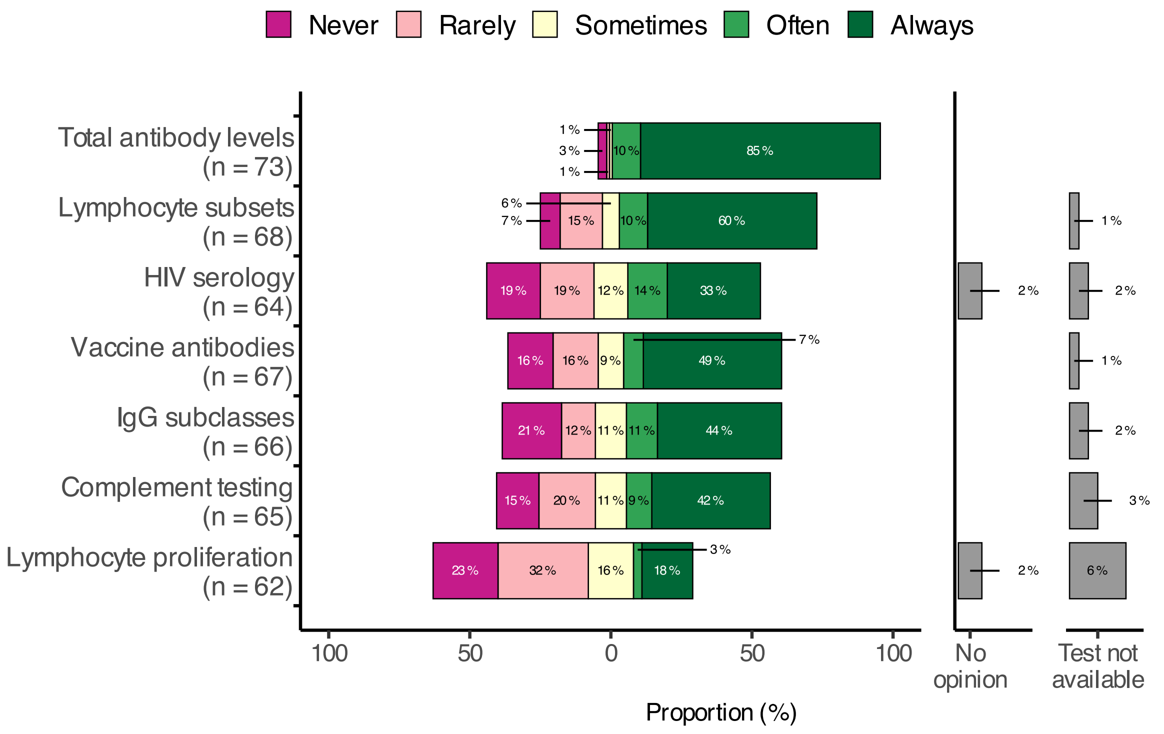

Details on immunological tests

When to test vaccine antibodies		Vaccine failure **25%** n = 55	Always **73%** n = 55	
Complement test to perform		C3/C4 **66%** n = 53	AP50 **43%** n = 53	CH100 **43%** n = 53
When to perform HIV test	Mum HIV infected **34%** n = 50	No test in pregnancy **28%** n = 50	High-prev country **34%** n = 50	Always **58%** n = 50
Would you do genetic testing?	No **75%** n = 148	Abnormal immunology **20%** n = 148	Normal immunology **3%** n = 148	Yes **3%** n = 148

Additional tests

In addition, 5 respondents requested a dihydrorhodamin test. Two asked for Panton-Valentine leukocidin testing in the S. aureus. One respondent each asked for a full blood count, mannose binding lectin, and MYD88.

Scenario 5

Case vignette

An 8-year-old girl presents with staphylococcal sepsis and necrotizing pneumonia. She requires inotropes and ventilation but eventually recovers. She is up-to-date with her vaccines. This is the second episode of an invasive infection. At the age of 3 years she had presented with E. coli bacteraemia. The family history is uneventful.

92% n = 146

Percentage performing immunological work-up

Immunological testing respondents would perform

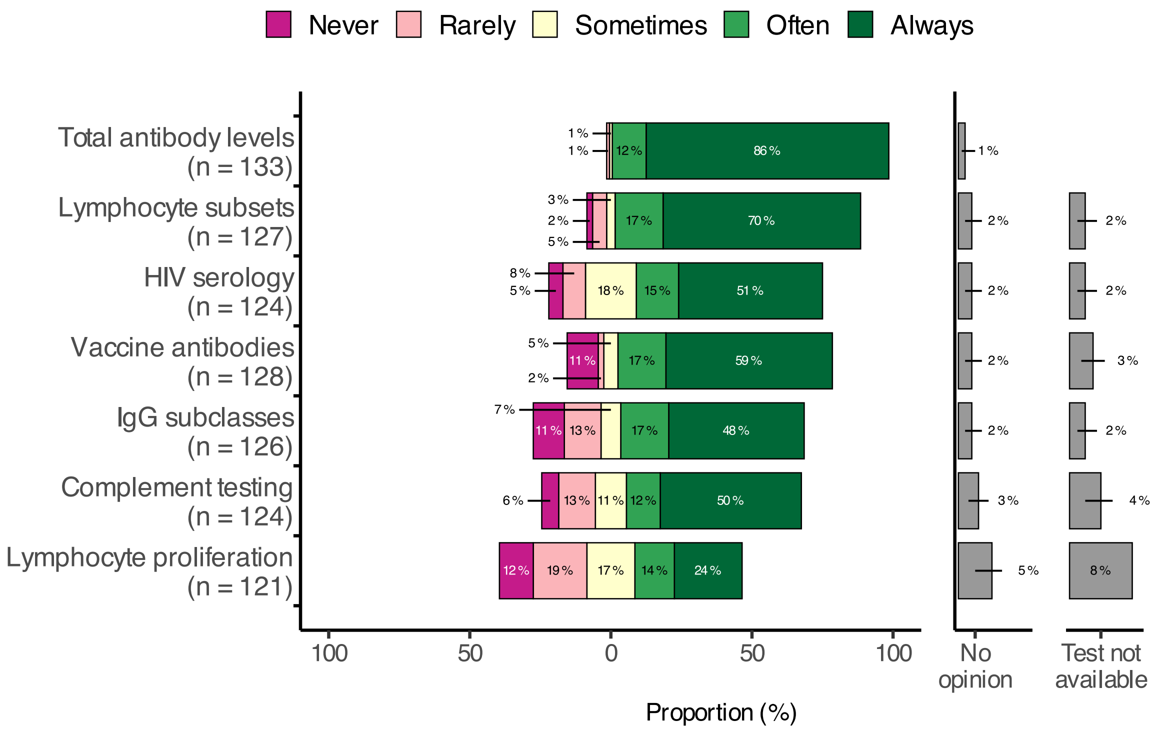

Details on immunological tests

When to test vaccine antibodies		Vaccine failure **16%** n = 107	Always **82%** n = 107	
Complement test to perform		C3/C4 **72%** n = 107	AP50 **57%** n = 107	CH100 **58%** n = 107
When to perform HIV test	Mum HIV infected **28%** n = 114	No test in pregnancy **22%** n = 114	High-prev country **25%** n = 114	Always **64%** n = 114
Would you do genetic testing?	No **30%** n = 144	Abnormal immunology **37%** n = 144	Normal immunology **11%** n = 144	Yes **22%** n = 144

Additional tests

In addition, 7 respondents requested a dihydrorhodamin test. Two asked for IgE. One respondent each asked for a full blood count, mannose binding lectin, and an abdominal ultrasound.

Scenario 6
